# Supplementary material for: Food and Non-Food-Related Behavior across Settings in Children with Prader–Willi Syndrome
Source: Genes (Basel). 2020 Feb 17;11(2):204. doi: 10.3390/genes11020204 (PMC7074075; doi:10.3390/genes11020204)
Supplement: Supplementary file 1 [file genes-11-00204-s001.docx]

Table S1. Characteristics of GAIB Study Participants with 1 vs. 2 Assessments

| **Characteristic** | **Category** | **Parent and Teacher (N=49)** | **Only One Assessment (N=51)** | **p Value** |
| --- | --- | --- | --- | --- |
| Age at assessment | Mean (n=100) | 8.9 (4.0) | 9.6 (4.5) | 0.42 |
| Male |  | 26 (53.1%) | 22 (43.1%) | 0.32 |
| White Race | Yes | 42 (85.7%) | 40 (78.4%) | 0.60 |
|  | No | 5 (10.2%) | 7 (13.7%) |  |
|  | Unknown | 2 (4.1%) | 4 (7.8%) |  |
| Mother Graduated from College | Yes | 23 (46.9%) | 24 (47.1%) | 0.16 |
|  | No | 20 (40.8%) | 14 (27.5%) |  |
|  | Unknown | 6 (12.2%) | 13 (25.5%) |  |
| Deletion | Yes | 27 (55.1%) | 28 (54.9%) | 0.91 |
|  | No | 13 (26.5%) | 15 (29.4%) |  |
|  | Unknown | 9 (18.4%) | 8 (15.7%) |  |
